# Comprehensive analysis of lncRNA-mRNA co-expression patterns identifies immune-associated lncRNA biomarkers in ovarian cancer malignant progression

**DOI:** 10.1038/srep17683

**Published:** 2015-12-03

**Authors:** Qiuyan Guo, Yan Cheng, Tian Liang, Yanan He, Chengcheng Ren, Liyuan Sun, Guangmei Zhang

**Affiliations:** 1 The First Affiliated Hospital of Harbin Medical University, Harbin 150001, Heilongjiang, China

## Abstract

Ovarian cancer (OV) is the most common and lethal gynecological tumor with a poor prognosis for women; however, the regulatory roles of the long non-coding RNAs (lncRNAs) in ovarian malignant progression are insufficiently understood. Here, we investigated the expression patterns of lncRNAs and mRNAs in the high-throughput molecular profiles of 399 OV patients and constructed a functional lncRNA-mRNA co-expression network across OV malignant progression. We found that two protective lncRNAs, RP11-284N8.3.1 and AC104699.1.1, were not only differentially expressed throughout the progression of malignant OV but were also independently predictive of the survival of patients with different OV stages. A functional analysis of the two lncRNAs predicted their roles in immune system activation and other anti-tumor processes in the OV microenvironment. Integrating these two lncRNAs into an OV risk model was able to significantly stratify patients into different risk groups. Overall, our analysis effectively provides insights into the lncRNA association with malignant OV progression. The two-lncRNA signature is a candidate biomarker for the prognosis of patients with OV and may enable a more accurate prediction of survival.

Ovarian cancer (OV) is the most lethal gynecological cancer and a common cause of cancer-related deaths in women globally^1^. During the asymptomatic stage of the disease, OV is characterized by the frequent development of metastases in the pelvic and abdominal cavity^2^. As a result, most patients have already developed metastases when they are first diagnosed. Despite advances in chemotherapy and surgical treatment, the extremely poor prognosis of OV remains unsatisfactory, with only a 30% five-year survival rate^3^. This high overall mortality rate is likely due to the lack of overt symptoms during the early stages of this disease (pathological stages I and II) and reliable early detection procedures. In the late stages (stages III and IV), ovarian tumors have often metastasized or spread to other organs, leading to a poor prognosis^4^. Therefore, it is important to understand the molecular mechanisms of OV development, to identify reliable markers of ovarian cancer, and to use these markers to predict clinical patient outcomes.

Long non-coding RNAs (lncRNAs), which are non-protein-coding transcripts longer than 200 nucleotides, are regulators that have been recently discovered in a wide range of biological functions^5^, such as chromatin modification and genomic imprinting^6^,^7^. In addition, emerging evidence indicates that lncRNAs play complex and extensive roles in cancer development and progression^8^. For example, lncRNA HULC has been shown to play an important role in tumorigenesis by acting as an endogenous sponge transcript^9^. LncRNA MALAT1 functions as an oncogene, and its expression correlates with metastases and survival in lung cancer^10^. LncRNA HOTAIR has a high expression level in metastatic breast cancer tumors, and the inhibition of HOTAIR expression may block tumor metastasis^11^. The over expression of HOTAIR is predictive of poor patient prognosis and promotes tumor metastasis in epithelial ovarian cancer^12^. However, these reports have provided only a limited understanding of lncRNAs, and the identification of cancer-related lncRNAs remains a challenging task. Thus, a ‘guilt by association’ strategy is always used to functionally characterize lncRNAs^13^. Co-expression models, which integrate protein-coding genes and lncRNAs, are constructed to study lncRNA functions in biological processes and cancers^13^,^14^. However, due to the lack of simultaneous profiling of lncRNA and mRNA expression levels across samples from different stages, few studies have reported the presence of an OV progression-associated lncRNA-mRNA regulatory network. RNA-sequencing data can be used to perform these analyses by obtaining whole-transcriptome shotgun sequences and detecting the less-abundant transcripts of mRNAs and lncRNAs^15^.

In this study, we used a multi-step approach to construct a functional lncRNA-mRNA regulatory network (OVLMN) that is associated with ovarian cancer malignant progression. Our comprehensive analyses used the high-throughput molecular profiles of 399 ovarian serous cystadenocarcinoma patients generated within The Cancer Genome Atlas (TCGA)^16^. A systematic analysis identified that lncRNAs exhibit specific topological characteristics in the OVLMN, indicating their association with coding-genes in the progression of malignant OV. Furthermore, we uncovered two lncRNAs, RP11-284N8.3.1 and AC104699.1.1, that were differentially expressed throughout the progression of malignant OV and can predict the survival of patients with different OV stages. Moreover, we found that these two protective lncRNAs were one-step neighbors in the OVLMN and may co-regulate OV progression. A functional analysis revealed that the two lncRNAs were co-associated with immune system activation and other anti-tumor processes in the OV microenvironment. Integrating these two lncRNAs into a risk model significantly stratified OV patients into different risk groups. This comprehensive analysis provided novel insights into lncRNA roles in malignant OV progression at the transcriptomic level. These results and conclusions can serve as important resources for future experimental dissection of lncRNAs in OV.

## Results

### Comprehensive construction of the OVLMN reveals specific patterns of OV-associated lncRNAs

**Figure 1:**
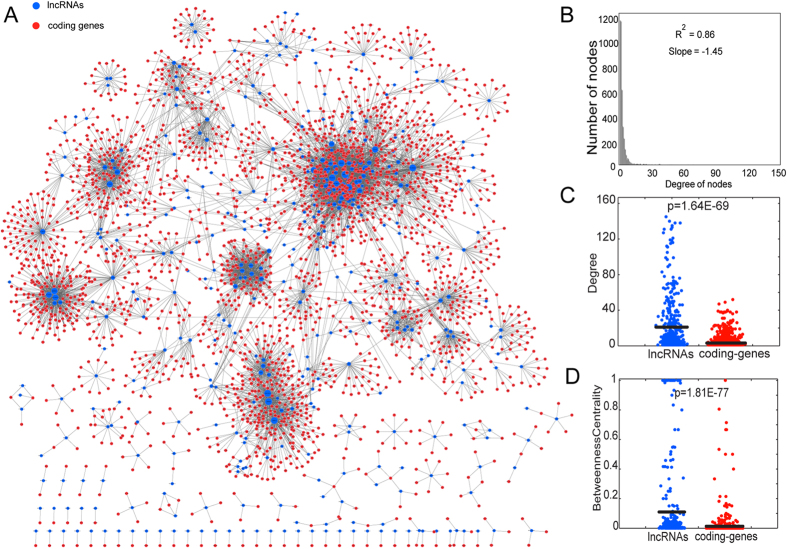
Illustrations of the OVLMN and its characteristics. (**A**) The OVLMN consisted of 8393 co-expression relationships between 399 lncRNAs and 2634 mRNAs. Blue circles denote lncRNAs, and red circles denote mRNAs. The node degree is indicated by the circle size. An edge represents a co-expression relationship between a lncRNA and an mRNA in the context of OV progression. (**B**) The degree distribution of the OVLMN is shown as an individual plot. Most of the nodes are poorly connected, and a few are relatively highly connected. The network reveals a power-law distribution with a slope of −1.46 and R^2^ = 0.86. (**C**) The LncRNA nodes have significantly higher degrees than the coding-gene nodes in the OVLMN. (**D**) The LncRNA nodes have significantly higher betweenness centrality than the coding-gene nodes in OVLMN.

We performed a multi-step approach to construct the OVLMN. A differential expression analysis was performed by comparing expression profiles of patients in stage III and IV with those in stage II to identify OV progression-associated lncRNAs and coding genes. In total, 536 lncRNAs and 9053 coding genes exhibited significant differential expression in stages III and IV versus stage II OV samples, suggesting widespread variation in both lncRNA and mRNA expression in malignant OV progression. We further investigated the co-expression relationships between the differentially expressed lncRNAs and coding genes. Significantly co-expressed lncRNA-mRNA pairs (P < 0.01) were identified and assembled into the OVLMN, which consisted of 8393 co-expression relationships between 399 lncRNAs and 2634 mRNAs (Fig. 1A). The major component (the maximum connected subgraph of the network) contained 295 lncRNAs and 2366 mRNAs. An examination of the node degrees in the OVLMN revealed a power-law distribution with a slope of −1.46 and R^2^ = 0.86 (Fig. 1B). These results indicate that the OVLMN is similar to many biological networks^17^, and it is well characterized by a core set of co-expression regulation principles in structure that distinguishes it from randomly generated networks. We then considered the topological characteristics of the OVLMN, such as node degree and betweenness centrality. A higher degree indicated that the nodes were likely to be hubs and therefore involved in more competing interactions, whereas a higher betweenness centrality (BC) implied that they acted as bridges connecting different network components and controlling communication. We found that the lncRNA nodes had significantly higher degrees and BCs than the coding-gene nodes (Fig. 1C,D). Moreover, the top 50 large degree nodes and top 20 BC nodes were all lncRNAs. These results indicate that although not coding for a protein, lncRNAs exhibit more specific topological characteristics than coding genes in the OVLMN throughout malignant OV progression.

### Two hub lncRNAs in the OVLMN were associated with OV patient prognosis

**Figure 2:**
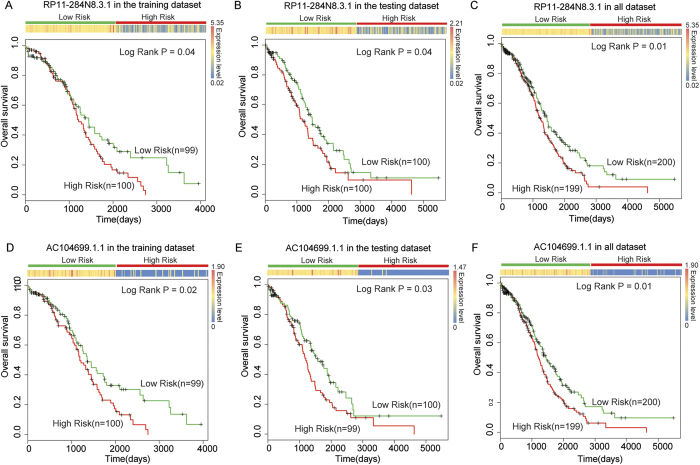
The use of lncRNAs to predict the clinical outcomes of OV patients. The expression profile of the lncRNAs is shown in the top panel. The corresponding Kaplan-Meier survival plot (bottom) of the two patient subgroups. Patients who showed no progression or who were still alive at the time of the last follow-up were censored (+). Survival days are shown along the *x*-axis. Overall survival rates are shown along the *y-*axis. LncRNA RP11-284N8.3.1 was able to distinguish patients with different clinical outcomes in (**A**) the training dataset, (**B**) the testing dataset and (**C**) the combined dataset. LncRNA AC104699.1.1 was able to distinguish patients with different clinical outcomes in (**D**) the training dataset, (**E**) the testing dataset and (**F**) the combined dataset.

Based on the specific patterns of the lncRNAs in the OVLMN, we hypothesized that these lncRNAs might be involved in malignant OV development and influence progression. Thus, we tested the efficiency of the hub lncRNAs as prognostic signatures. In biological networks, hubs are commonly defined as the top 15% of the nodes by degree^18^,^19^. In this study, as a more stringent threshold, we defined hubs as the top 5% (top 151) of the nodes by degree. These 151 hub nodes contained 136 lncRNAs that co-expressed with approximately 50% of the nodes in the OVLMN, implying the centrality of these hub lncRNAs. Subsequently, we examined whether these hub lncRNAs were associated with the survival of OV patients. We performed a univariate Cox regression analysis based on the expression value of these lncRNAs and found nine lncRNAs had significant effects on patient survival (Supplementary Table S1). To further test whether these nine lncRNAs could be used as OV prognosis biomarkers, we built a risk score model, as described in the methods section. In total, 399 OV patients were randomly assigned to the training set (n = 199) or the testing set (n = 200). There were no significant differences in clinical characteristics between the two sets(Table 1). After a survival analysis, we found that two lncRNAs, RP11-284N8.3.1 (ENSG00000259834) and AC104699.1.1 (ENSG00000224220), could significantly divide patients into high- and low-risk groups in the training set (Fig. 2A,D). Next, we validated each of the two lncRNAs in the testing set using the same cut-off point identified in the training set. Similarly, patients were also divided into two different risk groups (Fig. 2B,E). We then used RP11-284N8.3.1 and AC104699.1.1 as prognosis biomarkers to distinguish all 399 OV patients and obtained significant classification results (Fig. 2C,F).

**Figure 3:**
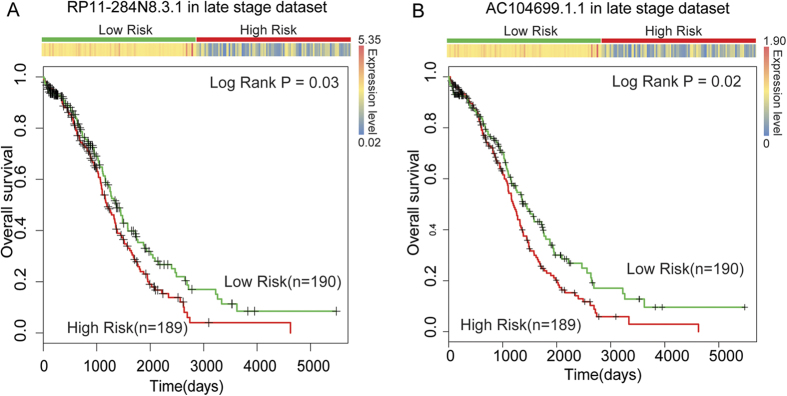
The use of lncRNAs to predict the clinical outcomes of late-stage OV patients. LncRNAs RP11-284N8.3.1 (**A**) and AC104699.1.1 (**B**) were able to significantly distinguish late-stage OV patients with different clinical outcomes.

As patients in the late stages of OV often have a poor survival rate, we tested whether these two lncRNAs could be used as prognosis signatures for OV patients in stages III and IV. After survival analysis, we found that both RP11-284N8.3.1 and AC104699.1.1 could significantly divide late-stage patients into high- and low-risk groups (Fig. 3). In the univariate Cox regression analyses, both RP11-284N8.3.1 and AC104699.1.1achieved a regression coefficient with a minus sign (Supplementary Table S1), which indicates that increased expression is associated with a decreased risk of survival. From Fig. 2A–F, we found that the predicted low-risk groups always had higher expression levels of lncRNAs RP11-284N8.3.1 and AC104699.1.1 than the high-risk groups. This observation was consistently found in survival analyses of the training, testing, late-stage and overall OV patients. These results indicate that lncRNAs RP11-284N8.3.1 and AC104699.1.1are protective factors throughout malignant OV progression.

Moreover, we performed additional analyses to test whether the lncRNAs are predictive of progression-free survival. In a Cox regression analysis (Supplementary Table S2), we found that both RP11-284N8.3.1 (P = 0.01) and AC104699.1.1 (P = 0.02) were significantly associated with patient progression-free survival. After a Kaplan-Meier survival analysis, we found that only lncRNA RP11-284N8.3.1 (P = 4.07E-3) can significantly divide the 399 OV patients into two groups with different progression-free survival rates (Supplementary Figure S1).

### The two protective lncRNAs involved in immune- and cell cycle-associated processes

**Figure 4:**
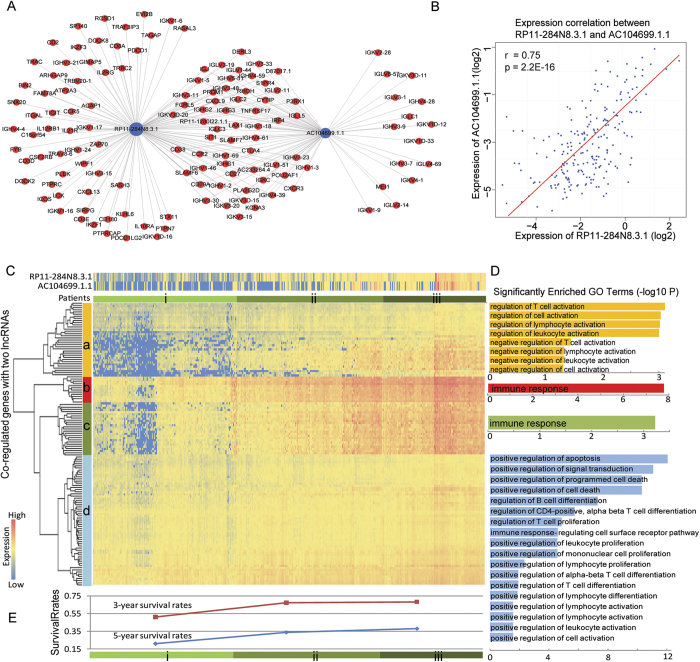
Comprehensive analysis of the function and expression of the two lncRNA subnetworks. (**A**) LncRNAs RP11-284N8.3.1 and AC104699.1.1 are one-step neighbors in the OVLMN and co-regulate 56 coding genes, most of which are immunoglobulin genes. (**B**) The expression of these two lncRNAs was highly correlative(Pearson Correlation: r = 0.75, p = 2.2E-16) (**C**) The expression profiles of the two lncRNAs and their regulatory coding genes. Based on a two-dimensional hierarchical cluster analysis, the coding genes were grouped into four classes across the three groups of patients. (**D**) A functional enrichment analysis for each group of genes indicates the immune- and cell cycle-associated roles of biological processes. (**E**) The corresponding 3-year and 5-year survival rates of each group of patients. The survival rates increased from patients in group *i* to those of group *iii*.

Given that RP11-284N8.3.1 and AC104699.1.1are both OV protective factors, we predicted their functions based on their network contexts. We found that these two protective lncRNAs were one-step neighbors in the OVLMN and co-expressed with 56 coding genes, most of which were immunoglobulin genes (Fig. 4A). Moreover, the expression of these two lncRNAs was highly correlative (Pearson Correlation: r = 0.75, p = 2.2E-16; Fig. 4B). In this sub-network, lncRNA RP11-284N8.3.1 has the highest Pearson correlation coefficient (r = 0.93) with KCNA3 (Supplementary Figure S2). These observations indicate that RP11-284N8.3.1 and AC104699.1.1 may synergistically co-involved in malignant OV progression. To investigate the functions of these two lncRNAs, we used a ‘guilt by association’ strategy to study lncRNA functions^13^,^14^. We investigated the expression patterns of the two lncRNAs and their co-expressing coding-gene neighbors in the OVLMN. A hierarchical clustering analysis revealed specific gene expression clusters across different OV patient groups (Fig. 4C). Based on their expression, these genes could be generally classified into four different sets (a–d in Fig. 4C). The patients could be classified into three groups (i, ii or iii in Fig. 4C) with different gene expression patterns. A functional enrichment analysis based on GO Terms was performed for each of the four gene sets (Fig. 4D). The four gene sets significantly participated in immune- and cell cycle-associated terms, indicating that they might have important biological implications for OV oncogenesis. Gene set a was significantly enriched in the regulation of immune cell activation, such as T cell activation and lymphocyte activation. Gene sets b and c were significantly enriched in immune response genes. Gene set d was enriched in several tumor suppressive processes (such as the positive regulation of apoptosis and cell death) and a series of immune activation processes (such as T cell proliferation and differentiation). It has been well documented that the immune system can attack tumor cells and provide a natural defense against cancers^20^,^21^. Previous studies have indicated that OV patients’ clinical outcomes and five-year survival rates are associated with immune effector cells^22^, such as tumor-infiltrating lymphocytes and T cells. In Fig. 4C, the expression levels of RP11-284N8.3.1 and AC104699.1.1increased as the expression of these immune-associated gene sets increased from group i to iii. The 3-year and 5-year survival rates also increased with these gene sets. Details regarding the survival rates of the three groups are illustrated in Supplementary Figure S3. We found that patient group i had a significantly lower survival rate than those of group ii (P = 0.04) and iii (P = 9.61E-4). Correspondingly, group-i patients had significantly lower expression levels of gene sets a, b and c than those in group iii (Mann-Whitney*U*-test, P = 2.2E-16), indicating that the immune system was partially suppressed or poorly activated in the group-i patients. The lower expression level of gene set d (group i vs group iii, P = 1.6E-13) revealed the inhibition of primary adaptive immune responses (such as the differentiation and proliferation of immune cells) and some anti-tumor progression processes (such as cell death and apoptosis) in group-i patients, which had the lowest 3-year and 5-year survival rates (Fig. 4E). The group-iii patients with activated immune systems had the highest survival rate. These results suggest that lncRNAs RP11-284N8.3.1 and AC104699.1.1are both protective factors throughout malignant OV progression and are associated with the activation of the immune system and other anti-tumor processes in the OV microenvironment.

### Synergistic co-regulation of RP11-284N8.3.1 and AC104699.1.1 increases efficiency in prognostic performance

**Figure 5:**
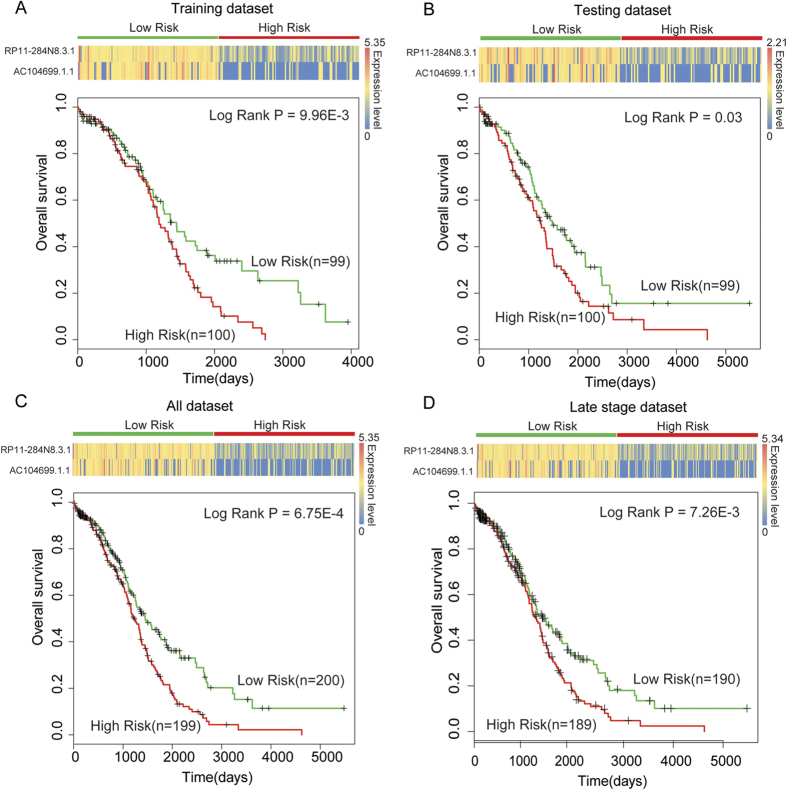
The integration of two lncRNAs predicts the clinical outcome of OV patients. The expression profiles of the lncRNAs are shown in the top panel. A corresponding Kaplan-Meier survival plot (bottom) of the two patient subgroups. The ITLS was able to distinguish patients with different clinical outcomes in (**A**) the training dataset, (**B**) the testing dataset, (**C**) the combined dataset and (**D**) the late-stage dataset. Patients who showed no progression or who were still alive at the time of the last follow-up were censored (+). Survival days are shown along the *x*-axis. Overall survival rates are shown along the *y*-axis.

Based on these analyses, we observed that RP11-284N8.3.1 and AC104699.1.1 are one-step neighbors, OV protective factors, and highly co-expressed in the OVLMN. These observations indicated that RP11-284N8.3.1 and AC104699.1.1 might have synergistic, co-functions throughout malignant OV progression. To further examine whether these two immune-associated lncRNAs could cooperatively contribute to the survival of OV patients, we integrated the expression levels of RP11-284N8.3.1 and AC104699.1.1 into a comprehensive risk score, as described in the Methods section. The integrated risk score was able to significantly divide patients into high- and low-risk groups in the training set (Fig. 5A). We then defined lncRNAs RP11-284N8.3.1 and AC104699.1.1 as an integrated two-lncRNA signature (ITLS) and validated the classification efficiency of the ITLS in the testing set using the same risk score cut-off point identified in the training set. Similarly, patients were also significantly divided into two different risk groups (Fig. 5B). We then used the ITLS as a prognosis biomarker to distinguish all 399 OV patients and achieved significant classification results (Fig. 5C). The log-rank P value of the ITLS (P = 6.75E-4) was more significant than the single use of either RP11-284N8.3.1 (P = 0.01) or AC104699.1.1 (P = 0.01). In late-stage OV, the ITLS was able to significantly divide late-stage patients into high- and low-risk groups (P = 7.26E-3). The log-rank P value of the ITLS in the late stage was also more significant than the single use of either lncRNA.

### ITLS is an independent prognosis biomarker

To further ascertain whether the risk signature comprised of the two lncRNAs is an independent predictor of OV patient survival, the prognostic association between our newly identified ITLS and other known clinical and pathological risk factors for malignant OV progression was assessed by univariate and multivariate analyses. Several clinicopathological factors, such as age, stage, histological grade and residual tumor diameter, were considered. Lymph node metastasis was not analyzed due to the large number of missing values. As expected, in addition to patient age, which is already a well-known risk factor, the ITLS was a significant risk factor for survival in a univariate analysis (P = 0.01; Table 2). A multivariate analysis further revealed that the ITLS remained an independent prognostic risk factor for OV patient survival (P = 0.02; Table 2). In addition, a multivariate analysis was separately performed for each lncRNA in the ITLS. We found that lncRNA RP11-284N8.3.1 was also an independent prognostic factor in OV (Supplementary Table S3 and S4).

## Discussion

LncRNAs are emerging as regulators in a wide range of biological functions^5^. These newly characterized regulators play complex and extensive roles in cancer development and progression^13^. At present, there is a lack of comprehensive databases that provide a resource for experimentally verified lncRNA functions. Experimental validation of roles for thousands lncRNAs is complex, expensive and laborious. Bioinformatics approach, such as co-expression analysis, is always performed to infer lncRNA functions. The expression profiles of protein-coding genes and lncRNAs are integrated into co-expression models to study lncRNA characteristics in different biological processes and cancers^13^,^14^. For example, Liao *et al.* have performed large-scale prediction of lncRNA functions in a coding-non-coding gene co-expression network^23^. They further developed a online web server, named ncFANs, to provide lncRNA annotations of human and mouse^24^. Guo *et al.* have developed a bi-colored (lncRNA and mRNA co-expression) network based global function predictor of lncRNAs^13^. Based upon these studies, we believe that our work will expand our knowledge of lncRNA associated malignant OV progression and play an important role as a pre-processing step to guide further ‘wet’ lab experimental designs. LncRNAs are more likely to be co-expressed with their neighboring coding-genes through cis-regulatory mechanisms^14^. According to Wang’s study^25^, some lncRNAs are co-expressed with their corresponding coding-genes through ceRNA theory. Other regulatory mechanisms involving transcription factors, DNA methylation, and copy number variations can also induce the co-expression of lncRNAs and protein-coding genes and further contribute to cancer pathology.

In ovarian cancer, which is the most lethal gynecological cancer and a common cause of cancer-related deaths in women globally^1^, few studies have reported the presence of ovarian cancer progression-associated lncRNA-mRNA co-expression networks due to the lack of simultaneous expression profiles of lncRNAs and mRNAs across samples from different OV stages. With the advancement of sequencing technology, RNA-sequence data can be used to perform these analyses by obtaining whole-transcriptome shotgun sequences and detecting the less-abundant mRNA and lncRNA transcripts^15^. However, there are limitations of this technology. RNA-sequence was not perfect in detecting gene expression when two genes were close to each in the genome. To test this effect, we investigated the genomic associations between lncRNA and coding-gene pairs in the network. Among the 8393 lncRNA-coding-gene pairs, we found that there were 1558 pairs locating in the same chromosome and only 53 pairs locating within 10 kb distance. 10 kb distance was used as a threshold to study co-expression effect between lncRNAs and their neighbour coding-genes^14^. Expression association and genomic location for the 8393 lncRNA-coding-gene pairs were illustrated in Figure S4.

In the present study, we investigated the expression patterns of lncRNAs and mRNAs and constructed a functional lncRNA-mRNA regulatory network for malignant ovarian cancer progression using the high-throughput molecular profiles of 399 OV patients generated within TCGA^16^. Through an analysis of tumor samples in different stages, we found that many lncRNAs were differentially expressed, indicating that lncRNAs may be associated with malignant OV progression. We hypothesized that these differentially expressed lncRNAs and coding genes were associated with malignant OV progression (development from early to late stages). Furthermore, we investigated the co-expression relationship between these differentially expressed lncRNAs and coding genes and built a co-expression network. This network can provide a global view of all possible lncRNA-coding gene expression associations based on the malignant OV background. As in previous studies, the topological structure of the co-expression network can help us to identify cancer prognostic signatures^26^. A similar strategy has been used in a previous study to identify miRNA risk signatures of malignant glioma progression^27^ and lncRNA signatures in other cancers^25^.

A systematic analysis of the OVLMN indicated that lncRNAs exhibit specific topological characteristics, implying that these lncRNAs are likely to be hubs and to control the communication of different network components. Based on these analyses, we uncovered two key lncRNAs, RP11-284N8.3.1 and AC104699.1.1, that were associated with most mRNAs in the OVLMN and may thus be involved in malignant OV progression and independently predictive of patient survival at different stages. In the co-expression network, we found these two protective lncRNAs were one-step neighbors in the OVLMN and co-expressed with many coding genes. Integrating these two lncRNAs into a risk model enabled significant stratification of the OV patients into different risk groups.

The correlation of lncRNA expression levels with the prognosis of patients with cancer has recently been reported for several malignant tumors^12^,^28^, such as hepatocellular carcinoma and breast cancer. Our discovery of a two-lncRNA signature in OV suggests that lncRNAs can be powerful predictors for the survival of cancer patients. In our study, the two-lncRNA signature identified in the training group exhibited similar prognostic value in both the test group and the overall cohort. We used the training set for detecting the lncRNA signature and further used the testing set for validation. The training and testing sets are sub-sets of the 399 OV cases and statistically independent from each other (Table 1). To test the overall predictive ability of these signatures, we further performed a survival analysis in all 399 OV patients. We found that the statistical P values for all 399 patients was more significant than in the training or testing datasets (Figs 2 and 5), indicating the classification efficiency for all OV patients. Thus, we believe that the prognostic power of this signature has a solid basis in patients with OV. Moreover, according to a recent report, the function of lncRNAs is more closely associated with their expression level compared with mRNAs, as they do not encode proteins^14^. As OV is highly metastatic, this cancer is diagnosed at an advanced stage in most cases. In our dataset, there were only 20 stage-II patients among the 399 OV cases. This small number of cases is a limitation of our study in the differential expression analysis. Although many differentially expressed lncRNAs and mRNAs can be identified by the DEGseq method^29^, some other risk lncRNAs or mRNAs may not be statistically significant due to the small number of stage-II patients. As the number of cancer cases in the TCGA project continues to grow, this bias will be addressed in future analyses.

In summary, our comprehensive analyses provided novel knowledge of lncRNAs at the transcriptomic level during malignant OV progression. These results and conclusions may serve as important resources for future experimental dissections of lncRNAs in OV.

## Methods

### Genome-wide RNA-sequencing data of mRNAs and lncRNAs in ovarian cancer

The mRNA and lncRNA expression dataset was derived from the study of Akrami *et al.*^30^, which identified 15,977 and 10,419 mRNAs and lncRNAs, respectively, from the TCGA OV RNA-sequencing dataset (available at http://www.larssonlab.org/tcga-lncrnas/). The annotations relied on the coding/non-coding classification provided by the GENCODE/Ensembl pipeline and considered as lncRNAs those genes that exclusively produce transcripts of the’antisense’, ‘lincRNA’, ‘non_coding’ and ‘processed_transcript’ types. This strategy has been used in previous studies to identify lncRNAs^25^,^30^. Genes producing non-coding mature transcripts shorter than 200nt were excluded. RPKM values were calculated using TCGA raw RNA-sequencing libraries in the BAM file format.

### Clinical characteristics of patients

Clinical and pathological data pertaining to the patients with OV were retrieved from the TCGA data portal. Staging and grading was performed in accordance with the criteria of the International Federation of Gynecologists and Obstetricians (FIGO) and the World Health Organization (WHO).The detailed clinicopathological characteristics of the patients are summarized in Table 1.In total, 399 samples (including 20 stage-II, 318 stage-III and 60 stage-IV patients) with clinical follow-up information were retained for further analysis.

### Identification of malignant OV progression-associated mRNAs and lncRNAs

We performed differential expression analysis by comparing mRNA and lncRNA expression in stage-III or stage-IV patients with those in stage-II patients to identify OV progression-associated mRNAs and lncRNAs. The DEGseq method^29^, whichis an R package to identify differentially expressed genes in RNA-sequencing data, was used in this step. The false discovery rate (FDR) was controlled at 0.01 thresholds (Benjamini and Hochberg algorithm). After analysis, 9503 mRNAs and 536 lncRNAs were identified as associated with malignant OV progression.

### Co-expression analysis

To identify co-expressed lncRNA-mRNA pairs, Pearson correlation coefficients were calculated based on the expression value between every differentially expressed lncRNA and mRNA pair. The threshold of Pearson correlation coefficients was set to >0.5, and the corresponding FDR was set to <0.01. Finally, 8393 co-expression relationships between 399 lncRNAs and 2634 mRNAs were identified.

### Development of the risk score model

To construct and validate the lncRNA signatures that predict the survival of OV patients, patients were randomly assigned to a training data set or a test data set. The two sample subsets were required to have the same size and have no significant difference in clinical characteristics (Table 1). As a more stringent threshold than used in previous studies^31^,^32^, the hub lncRNAs defined as the top 5% (top 151) of the nodes by degree in the OVLMN were considered as candidate signatures. A univariate Cox regression analysis was used to evaluate the association between survival and the expression level of each hub lncRNA. In the Cox regression model, a plus sign in the regression coefficient (RC) indicated that increased expression is associated with an increased risk of survival (risk lncRNAs). Conversely, a minus sign indicated that increased expression is associated with a decreased risk of survival (protective lncRNAs). After the univariate Cox regression analysis, a risk score formula was constructed that integrated both the strength and positive/negative association between each lncRNA and survival. The risk score for each patient was calculated according to the linear combination of the lncRNA expression values weighted by the RC from the univariate Cox regression analysis:


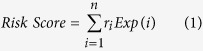


where *r*_*i*_ is the Cox RC of lncRNA *i* from the training set, and *n* is the number of testing lncRNAs. *Exp*(*i*) is the expression value of lncRNA *i* in a corresponding patient. The median risk score was used as the cut-off to classify the training dataset into high- and low-risk groups. Patients in the high-risk group were expected to have poor survival outcomes. Conversely, patients in the low-risk group were expected to have high survival outcomes. This cut-off point was further applied to the lncRNA expression data in the test set to divide the patients into high- and low-risk groups.

### Survival analysis

A Kaplan-Meier survival analysis was performed for the two classified groups of patients, and statistical significance was assessed using the log-rank test (P < 0.05). All analyses were performed on the R 3.1.0 framework.

### Betweenness centrality

BC is a measure of a node’s centrality in a network and is equal to the number of shortest paths from each node to all others that pass through this node; as such, it reflects the amount of control that a node exerts over the interactions of other nodes in the network. The BC of a node *n* is given by the following expression:


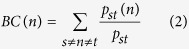


where *p*_*st*_ is the total number of shortest paths from node *s* to node *t*, and *p*_*st*_(*n*) is the number of these paths that passes through *n*.

### Functional enrichment analysis

We used a hypergeometric test to calculate the enrichment significance based on Gene Ontology (GO) terms. If the whole genome has a total of N genes, of which K are involved in the function category under investigation, and the set of interesting target genes for analysis has a total of M genes, of which x are involved in the same function category, then the P value can be calculated to evaluate the enrichment significance for that function category as follows:


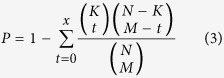


Significantly enriched GO terms were defined as FDR<0.05.

## Additional Information

**How to cite this article**: Guo, Q. *et al.* Comprehensive analysis of lncRNA-mRNA co-expression patterns identifies immune-associated lncRNA biomarkers in ovarian cancer malignant progression. *Sci. Rep.* **5**, 17683; doi: 10.1038/srep17683 (2015).

## Supplementary Material

Supplementary Information

## Figures and Tables

**Table 1:** Clinicopathologic characteristics of ovarian cancer patients (n = 399).

Characteristics	All patients n = 399	Number of patients	Test set n = 200	P
		Training set n = 199		
Stage				0.59^a^
II	20	11	9	
III	318	161	157	
IV	61	27	34	
Age				0.18^b^
Mean ± SD	59.54 ± 11.35	58.77 ± 11.62	60.31 ± 11.05	
Range	30–87	34–87	30–84	
Histological grade				0.90^a^
GX	7	4	3	
G1	1	0	1	
G2	46	22	24	
G3	344	173	171	
G4	1	0	1	
Residual tumor diameter (cm)				0.84^a^
<1	298	150	148	
>=1	101	49	52	
Lymph node metastasis				0.73^a^
Present	102	48	54	
Absent	54	26	28	
Unknown	243	125	118	
Survival (month)				0.62^b^
Mean ± SD	34.35 ± 27.70	33.66 ± 25.84	35.02 ± 29.48	
Range	0.30–182.70	0.30–131.77	0.30–182.70	
State				0.99^a^
Living	173	86	87	
Death	226	113	113	

^a^P-values were determined using chi-square test or Fisher’s exact test when appropriate.

^b^P-values were determined using Student’s t-test.

**Table 2:** Univariate and multivariate analysis of clinicopathological factors and ITLS in OV malignant progression.

Variables	Univariate analysis			Multivariate analysis		
	HR (95% CI)	Coefficient	P	HR (95% CI)	Coefficient	P
Stage	1.309 (0.9817–1.745)	0.2692	0.07	1.205 (0.8918–1.629)	0.186764	0.22
Age	1.016 (1.004–1.028)	0.0159	**0.01**	1.017 (1.0041–1.029)	0.016387	**8.81E–3**
Histological grade	1.056 (0.8052–1.385)	0.0546	0.69	1.061 (0.8083–1.392)	0.058934	0.67
Residual tumor diameter (cm)	1.337 (1.001–1.785)	0.2903	**0.04**	1.202 (0.8937–1.617)	0.184	0.22
ITLS	1.768 (1.102–2.386)	0.5699	**0.01**	1.737 (1.0808–2.790)	0.55188	**0.02**
