# Supplementary material for: Comprehensive analysis of lncRNA-mRNA co-expression patterns identifies immune-associated lncRNA biomarkers in ovarian cancer malignant progression
Source: Sci Rep. 2015 Dec 3;5:17683. doi: 10.1038/srep17683 (PMC4668366; doi:10.1038/srep17683)
Supplement: Supplementary Information [file srep17683-s1.pdf]

Supplementary Information

Comprehensive analysis of lncRNA-mRNA co-expression patterns identifies immune-associated lncRNA biomarkers in ovarian cancer malignant progression

Qiuyan Guo, Yan Cheng, Tian Liang, Yanan He, Chengcheng Ren, Liyuan Sun, Guangmei Zhang

Inventory of Supplementary Information

1. Supplementary Table S1
2. Supplementary Table S2
3. Supplementary Table S3
4. Supplementary Table S4
5. Supplementary Figure S1
6. Supplementary Figure S2
7. Supplementary Figure S3
8. Supplementary Figure S4

Supplementary Table S1. Univariate analysis of lncRNAs expression in OV malignant progression.

	Univariate analysis		
**Variable**	**HR (95%CI)**	**Coefficient**	**P-value**
PSMB8-AS1	0.970(0.941-0.999)	-0.0308	0.04
AC104699.1.1	0.444(0.214-0.920)	-0.8117	0.02
GS1-124K5.11.1	1.135(1.017-1.267)	0.1265	0.02
GAS5	1.002(1.000-1.267)	0.0022	0.04
RP11-320G24.1.1	0.621(0.416-0.928)	-0.4756	0.02
ZFHX4-AS1	1.485(1.217-1.812)	0.3958	9.80E-05
AL928768.3.1	0.298(0.122-0.724)	-1.2117	7.50E-03
RP11-284N8.3.1	0.656(0.458-0.940)	-0.4211	0.02

Supplementary Table S2. Univariate analysis of lncRNAs expression for progression-free survival.

	Univariate analysis		
**Variable**	**HR (95%CI)**	**Regression coefficient**	**P-value**
RP11-284N8.3.1	0.649(0.461-0.914)	-0.4317	0.01
AC104699.1.1	0.424(0.215-0.836)	-0.8573	0.02

Supplementary Table S3. Multivariate analysis of clinicopathological factors and RP11-284N8.3.1 in OV malignant progression

	Multivariate analysis		
**Variable**	**HR (95%CI)**	**Regression coefficient**	**P-value**
Stage	1.165(0.861-1.576)	0.153	0.32
Age	1.016(1.004-1.029)	0.016	0.01
Histological grade	1.069(0.813-1.404)	0.066	0.63
Residual tumor diameter(cm)	1.326(1.103-1.595)	0.282	2.72E-03
RP11-284N8.3.1	0.658(0.456-0.951)	-0.418	0.03

Supplementary Table S4. Multivariate analysis of clinicopathological factors and AC104699.1.1 in OV malignant progression

	Multivariate analysis		
**Variable**	**HR (95%CI)**	**Regression coefficient**	**P-value**
Stage	1.177(0.869-1.593)	0.163	0.29
Age	1.015(1.003-1.028)	0.016	0.01
Histological grade	1.054(0.803-1.384)	0.053	0.7
Residual tumor diameter(cm)	1.314(1.092-1.581)	0.273	3.77E-03
AC104699.1.1	0.497(0.239-1.033)	-0.699	0.06

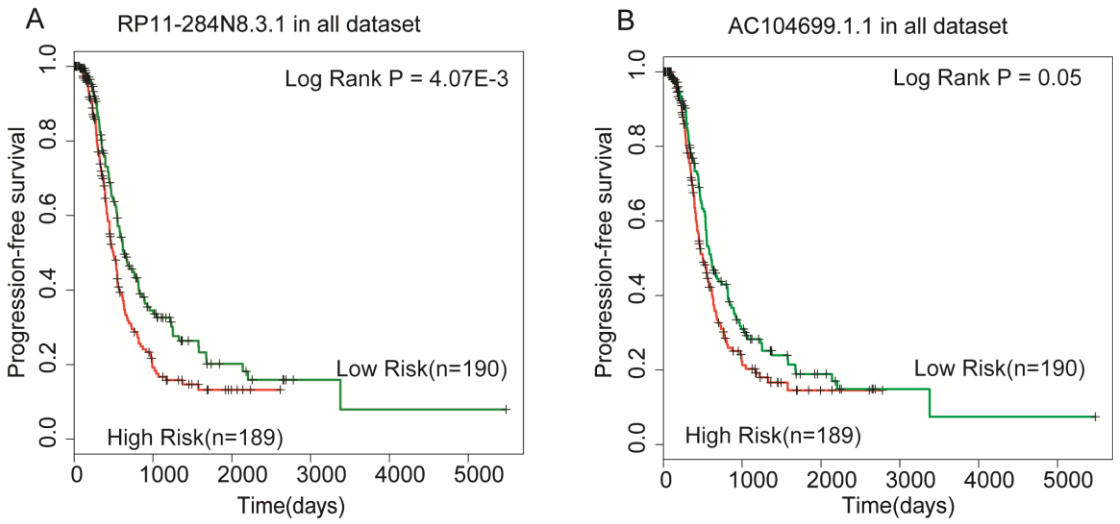

Figure S1. The use of lncRNAs to predict the progression-free survival of OV patients.

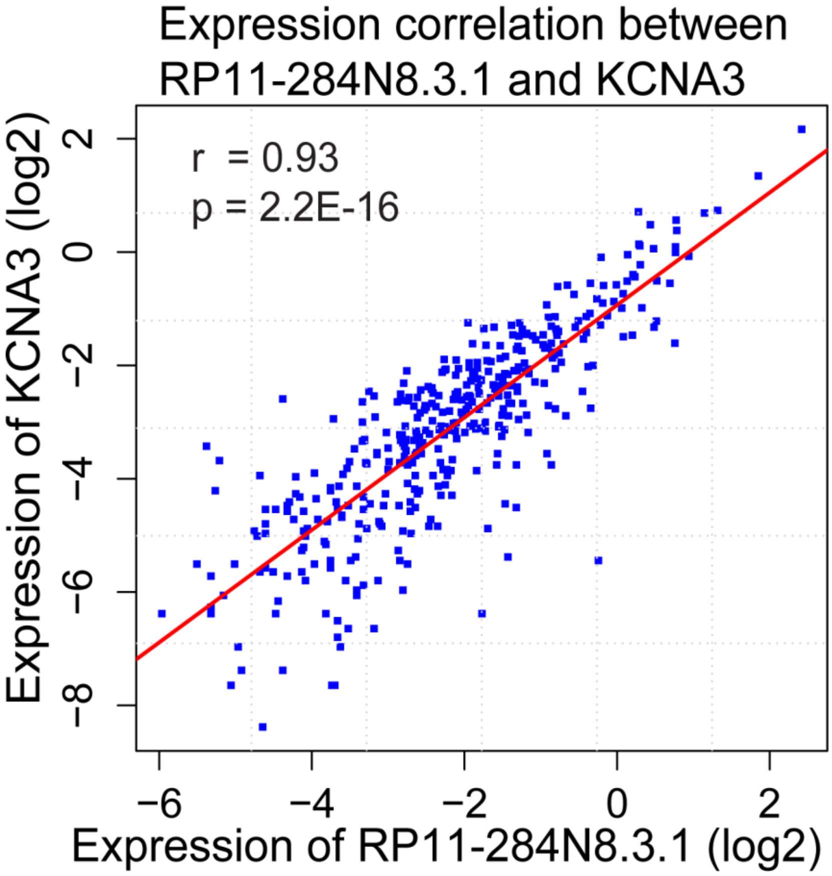

Figure S2. The expression illustration and correlation between RP11-284N8.3.1 and KCNA3.

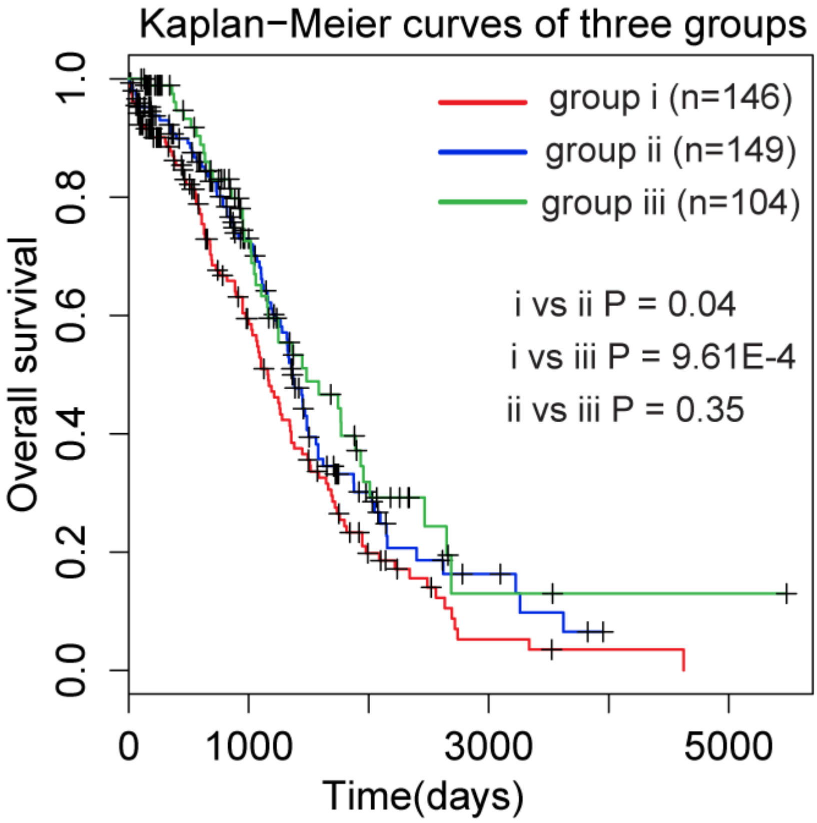

Figure S3. Detail information of survival rates for each of the three patient groups.

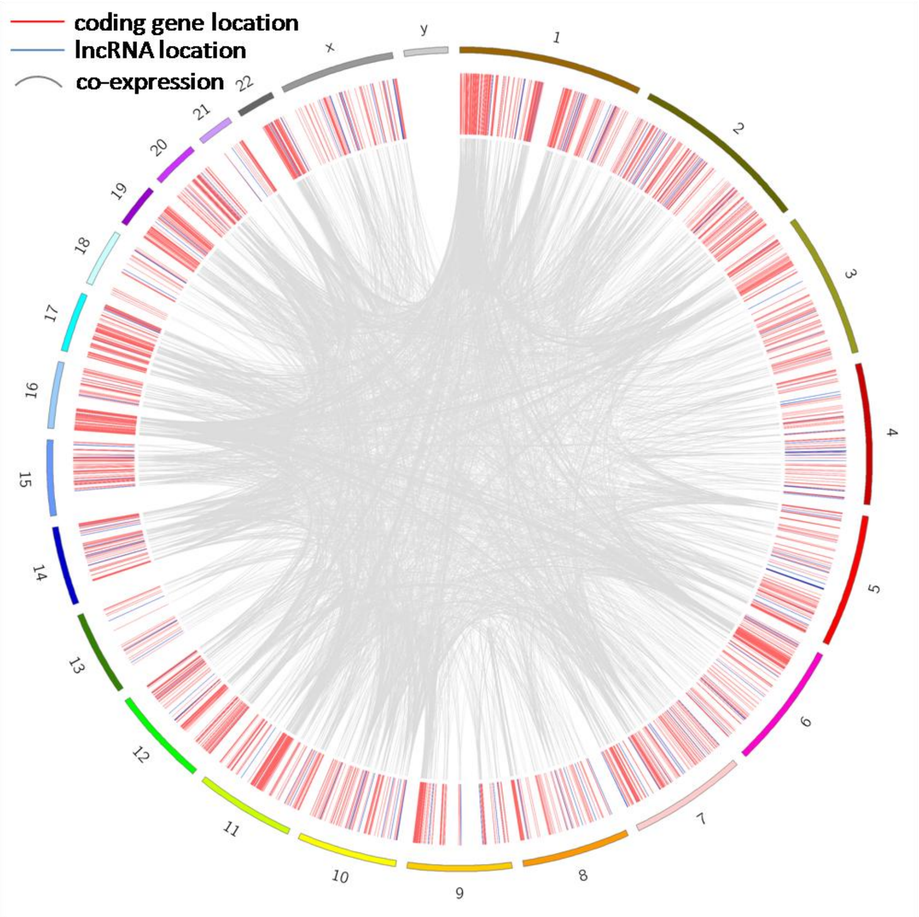

Figure S4. A genome-wide map of 8393 lncRNA-coding-gene pairs based on Circos-style visualization. Outer circles indicate human chromosomes. Genomic locations of lncRNAs and coding-genes were illustrated as blue and red bars. Grey arc lines within the circle denote pairs of co-expression interactions.
